# Correction: Cultivation Potential Projections of Breadfruit (*Artocarpus altilis*) Under Climate Change Scenarios Using an Empirically Validated Suitability Model Calibrated in Hawai’i

**DOI:** 10.1371/journal.pone.0241547

**Published:** 2020-10-28

**Authors:** The PLOS ONE Staff

Fig 5a is a duplicate of Fig 4b. The authors have provided a corrected version here. The publisher apologizes for the error.

**Fig 5:**
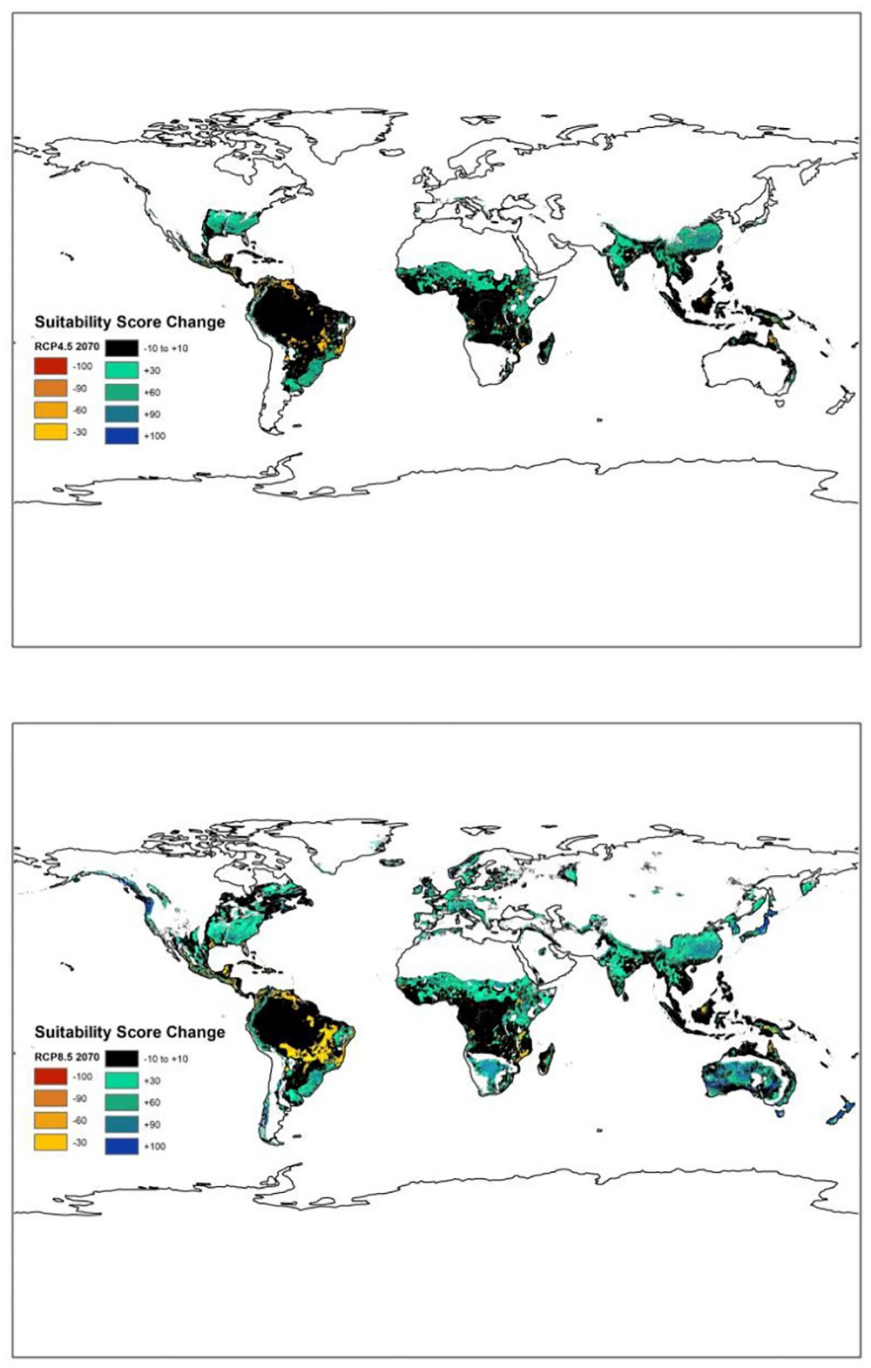
Changes (increase/decrease) in breadfruit suitability over the next 50 years under (top) RCP 4.5 and (bottom) RCP 8.5.
