# Supplementary material for: Cultivation potential projections of breadfruit (Artocarpus altilis) under climate change scenarios using an empirically validated suitability model calibrated in Hawai’i
Source: PLoS One. 2020 May 22;15(5):e0228552. doi: 10.1371/journal.pone.0228552 (PMC7244131; doi:10.1371/journal.pone.0228552)
Supplement: Attachment — Submitted filename: PONE-D-19-26938_reviewer.pdf [file pone.0228552.s001.pdf]

PLOS ONE

Growing Global Potential of Breadfruit Cultivation under Climate Change Scenarios

--Manuscript Draft--

Manuscript Number:	PONE-D-19-26938
Article Type:	Research Article
Full Title:	Growing Global Potential of Breadfruit Cultivation under Climate Change Scenarios
Short Title:	Modeling Current and Future Breadfruit Habitat
Corresponding Author:	Noa Kekuewa Lincoln, PhD<br>University of Hawaiʻi at Mānoa<br>Honolulu, Hawaiʻi UNITED STATES
Keywords:	breadfruit; artocarpus altilis; climate change; crop; suitability; geospatial; modeling; forecast; RCP 4.5; RCP 8.5; hawaii
Abstract:	Humanity faces significant challenges to agriculture and human nutrition, and changes in climate are predicted to make such challenges greater in the future. Neglected and underutilized crops may play a role in mitigating and addressing such challenges. Breadfruit, as a long-lived tree crop that is a nutritious, carbohydrate-rich staple is a priority crop in this regards. A fuzzy-set modeling approach was applied and refined for breadfruit using Hawai'i as a model system, with over 1,200 naturalized trees used to calibrate a habitat suitability model and 56 producer sites used to validate the model. The parameters were then applied globally. All 17 GCMs were used to drive the global breadfruit model for the RCP 4.5 and RCP 8.5 global climate projections for 2070. Overall, breadfruit suitability increases in area and in quality, with larger increases occurring on the more extreme scenario. Current producing regions largely remain unchanged in both scenarios, indicating relative stability of production potential in current growing regions. Breadfruit, and other tropical staples, be present strong opportunities for cultivation risk management strategies moving forward.
Order of Authors:	Kalisi Mausio
	Tomoaki Miura
	Noa Kekuewa Lincoln, PhD
Additional Information:	
**Question**	**Response**
**Financial Disclosure**<br><br>Enter a financial disclosure statement that describes the sources of funding for the work included in this submission. Review the submission guidelines for detailed requirements. View published research articles from *PLOS ONE* for specific examples.<br><br>This statement is required for submission and **will appear in the published article** if the submission is accepted. Please make sure it is accurate.	NKL have received McIntire-Stennis (8038-MS), HATCH (8035-H) and Western SARE (SW17-050) funding that has supported this project. The funders had no role in study design, data collection and analysis, decision to publish, or preparation of the manuscript.

Powered by Editorial Manager® and ProduXion Manager® from Aries Systems Corporation

**Unfunded studies**<br>Enter: *The author(s) received no specific funding for this work.*<br><br>**Funded studies**<br>Enter a statement with the following details:<br>• Initials of the authors who received each award<br>• Grant numbers awarded to each author<br>• The full name of each funder<br>• URL of each funder website<br>• Did the sponsors or funders play any role in the study design, data collection and analysis, decision to publish, or preparation of the manuscript?<br>• **NO** - Include this sentence at the end of your statement: *The funders had no role in study design, data collection and analysis, decision to publish, or preparation of the manuscript.*<br>• **YES** - Specify the role(s) played.<br><br>* typeset	
**Competing Interests**<br><br>Use the instructions below to enter a competing interest statement for this submission. On behalf of all authors, disclose any competing interests that could be perceived to bias this work—acknowledging all financial support and any other relevant financial or non-financial competing interests.<br><br>This statement **will appear in the published article** if the submission is accepted. Please make sure it is accurate. View published research articles from *PLOS ONE* for specific examples.	The authors have declared that no competing interests exist.

**NO authors have competing interests**<br><br>Enter: *The authors have declared that no competing interests exist.*<br><br>**Authors with competing interests**<br><br>Enter competing interest details beginning with this statement:<br><br>*I have read the journal's policy and the authors of this manuscript have the following competing interests: [insert competing interests here]*<br><br>* typeset	
**Ethics Statement**<br><br>Enter an ethics statement for this submission. This statement is required if the study involved:<br><br>• Human participants<br>• Human specimens or tissue<br>• Vertebrate animals or cephalopods<br>• Vertebrate embryos or tissues<br>• Field research<br><br>Write "N/A" if the submission does not require an ethics statement.<br><br>General guidance is provided below. Consult the submission guidelines for detailed instructions. **Make sure that all information entered here is included in the Methods section of the manuscript.**	N/A

Powered by Editorial Manager® and ProduXion Manager® from Aries Systems Corporation

**Format for specific study types**<br><br>**Human Subject Research (involving human participants and/or tissue)**<br>• Give the name of the institutional review board or ethics committee that approved the study<br>• Include the approval number and/or a statement indicating approval of this research<br>• Indicate the form of consent obtained (written/oral) or the reason that consent was not obtained (e.g. the data were analyzed anonymously)<br><br>**Animal Research (involving vertebrate animals, embryos or tissues)**<br>• Provide the name of the Institutional Animal Care and Use Committee (IACUC) or other relevant ethics board that reviewed the study protocol, and indicate whether they approved this research or granted a formal waiver of ethical approval<br>• Include an approval number if one was obtained<br>• If the study involved *non-human primates*, add *additional details* about animal welfare and steps taken to ameliorate suffering<br>• If anesthesia, euthanasia, or any kind of animal sacrifice is part of the study, include briefly which substances and/or methods were applied<br><br>**Field Research**<br><br>Include the following details if this study involves the collection of plant, animal, or other materials from a natural setting:<br>• Field permit number<br>• Name of the institution or relevant body that granted permission	
**Data Availability**<br><br>Authors are required to make all data underlying the findings described fully available, without restriction, and from the time of publication. PLOS allows rare exceptions to address legal and ethical concerns. See the PLOS Data Policy and FAQ for detailed information.	Yes - all data are fully available without restriction

Powered by Editorial Manager® and ProduXion Manager® from Aries Systems Corporation

A Data Availability Statement describing where the data can be found is required at submission. Your answers to this question constitute the Data Availability Statement and **will be published in the article**, if accepted.<br><br>**Important:** Stating 'data available on request from the author' is not sufficient. If your data are only available upon request, select 'No' for the first question and explain your exceptional situation in the text box.<br><br>Do the authors confirm that all data underlying the findings described in their manuscript are fully available without restriction?	
**Describe where the data may be found in full sentences. If you are copying our sample text, replace any instances of XXX with the appropriate details.**<br><br>• If the data are **held or will be held in a public repository**, include URLs, accession numbers or DOIs. If this information will only be available after acceptance, indicate this by ticking the box below. For example: *All XXX files are available from the XXX database (accession number(s) XXX, XXX.).*<br>• If the data are all contained **within the manuscript and/or Supporting Information files**, enter the following: *All relevant data are within the manuscript and its Supporting Information files.*<br>• If neither of these applies but you are able to provide **details of access elsewhere**, with or without limitations, please do so. For example:<br><br>*Data cannot be shared publicly because of [XXX]. Data are available from the XXX Institutional Data Access / Ethics Committee (contact via XXX) for researchers who meet the criteria for access to confidential data.*<br><br>*The data underlying the results presented in the study are available from (include the name of the third party*	Upon acceptance of publication all model codes and data layer files will be stored at GitHub for public access

and contact information or URL).<br>• This text is appropriate if the data are owned by a third party and authors do not have permission to share the data.<br><br>* typeset	
Additional data availability information:	Tick here if the URLs/accession numbers/DOIs will be available only after acceptance of the manuscript for publication so that we can ensure their inclusion before publication.

Powered by Editorial Manager® and ProduXion Manager® from Aries Systems Corporation

Manuscript

Click here to access/download;Manuscript;Breadfruit Model Paper_20190913.pdf

<PloS Biology>

<Research Article>

<For consideration of the special issue “Future Crops”>

Growing Global Potential of Breadfruit Cultivation under Climate Change Scenarios

Kalisi Mausio[1], Tomoaki Miura[2], Noa Lincoln[1,*]

[1]Tropical Plant and Soil Sciences, University of Hawai’i at Manoa, Honolulu, HI, 96822

[2]Natural Resources and Environmental Management, University of Hawai’i at Manoa, Honolulu, HI, 96822

*corresponding author: nlincoln@hawaii.edu

Growing Global Potential of Breadfruit Cultivation under Climate Change Scenarios

Abstract: Humanity faces significant challenges to agriculture and human nutrition, and changes in climate are predicted to make such challenges greater in the future. Neglected and underutilized crops may play a role in mitigating and addressing such challenges. Breadfruit, as a long-lived tree crop that is a nutritious, carbohydrate-rich staple is a priority crop in this regards. A fuzzy-set modeling approach was applied and refined for breadfruit using Hawai’i as a model system, with over 1,200 naturalized trees used to calibrate a habitat suitability model and 56 producer sites used to validate the model. The parameters were then applied globally. All 17 GCMs were used to drive the global breadfruit model for the RCP 4.5 and RCP 8.5 global climate projections for 2070. Overall, breadfruit suitability increases in area and in quality, with larger increases occurring on the more extreme scenario. Current producing regions largely remain unchanged in both scenarios, indicating relative stability of production potential in current growing regions. Breadfruit, and other tropical staples, ~~be~~ present strong opportunities for cultivation risk management strategies moving forward.

1. Introduction

Problem Statement

Humanity faces multiple challenges for the future of food production. By mid-21st century, the world population is expected to reach nine billion people with associated pressure on resources (Godfray et al., 2010), increasing demands on food and nutrition while rates of hunger and malnutrition are already on the rise (FAO et al., 2017), and changes in global weather resulting from alterations to the atmosphere and associated feedback processes are expected to negatively impact food yields, especially the major commodity crops that provide much of the

global food supply (Porter et al., 2014, 2017). Furthermore, global hunger and malnourishment has increased, largely correlated to poverty and therefore insufficient access to enough nutritious food (FAO et al., 2017; Mabhaudhi et al., 2016), with some 2 billion people suffering from micronutrient malnutrition (Buyck, 1993; Ramalingaswami, 1995). At the other end of the spectrum obesity, which is often attributed to global commodities that lack essential micro and macro-nutrients, is also on the rise globally (FAO et al., 2017). Increased pressure on food markets is expected to exacerbate existing issues of food security, nutrition, social equity, and economies (Godfray et al., 2010, Porter et al., 2014). In order to achieve the goals of eliminating hunger, improving food security and nutrition and promoting sustainable agriculture, the United Nations strongly believes in transforming and developing food systems that respond to or anticipate climate change (United Nations, 2015). With existing problems in our current food supply and compounding factors on the horizon, developing food production systems that emphasize resilience and nutrition is an essential strategy for mitigating issues.

Of the approximately 250-300,000 flowering plant species, at least 50,000 are edible (FAO, 1996, 1999). Of this, about 3,000 species are regularly exploited for food (Padulosi et al., 2002). Yet only three crops provide 60% of the world's calories (FAO, 2018) and 103 crops provide some 90% of the world's food production (Prescott-Allen and Prescott-Allen, 1990). This gap between the 3,000 species regularly utilized for food and the 103 dominant crops of the planet make up a vast, relatively untapped resource of crops commonly referred to as neglected and underutilized crops (NUS). Such crops are often ignored by researchers, policy makers and breeders (Padulosi, et al., 2002, 2013). NUS have a range of genetic resources that offer opportunities for adaptation, and by increasing agrobiodiversity farmers can enhance resilience to stressors related to climate change (Bacon et al., 2014). Such crops can further be an appropriate way to positively impact food and nutrition security, and diversification of diets through the utilization of NUS is imperative for food and nutrition security, especially with

expected impacts to current food systems and agricultural production methods. Due to a historical lack of research and investment into its growing conditions, agronomy, physiology, and cropping systems, it is likely that substantial gains in breadfruit yield could be made with relatively little effort into agronomic research and breeding (Sraffa, 2005; Willcox, 1954). However, multiple barriers exist to their widespread adoption. These barriers include loss of genetic diversity and traditional knowledge, undervaluation through lack of knowledge and research, poor competitiveness, and lack of infrastructure, policy support and investments (Padulosi et al., 2013). Therefore, more attention must be given to NUS on all levels - from cultivation to gastronomic uses - if they are to be useful in creating sustainable food systems for the future.

Brea[illegible]it

Breadfruit *(Artocarpus altilis*) is an underutilized traditional tree crop that grows in the tropics and sub-tropics, originally cultivated from the wild species ancestor bread nut (*Artocarpus camansi*) in Papua New Guinea (Ragone, 1997; Zerega et al., 2004). The crop is listed as one of the thirty-five priority crops in the multilateral system of the International Treaty on Plant Genetic Resources for Food and Agriculture for its potential to target food security and interdependence (FAO, 2009). The domestication of breadfruit transformed it into a unique, often seedless starchy staple crop (Jones et al., 2013a). The trees are high yielding, producing up to 10 t-ha-1 of fruit that is rich in carbo[illegible]rates, amino acids, fiber, vitamins, minerals including micronutrients such as iron and potassium (Jones et al., 2011; Jones et al., 2013b; Turi et al., 2015). All parts of the tree are used, from the wood to the latex to the male flowers, which are dried and burned to repel mosquitoes. Although exact fruit yield estimations are variable (see Lincoln et al., 2018a for a summary of yield), it is still evident that breadfruit has the potential to target issues surrounding food and nutrition security if specific cultivar and growing conditions are optimized.

Recent advances in propagation methods, scientific research and promotion of breadfruit have made breadfruit trees widespread; however, they are still underutilized (Ragone, 1997; Lincoln et al., 2018a). Today breadfruit is found throughout the tropics (Jones et al. 2013a, 2013b; Lincoln et al., 2018a). Breadfruit trees are low-maintenance, high producing perennial trees that has significant potential to contribute to building climate resilient food systems (McGregor et al., 2014, Bell and Taylor, 2015) in addition to its ability to sequester carbon, provide shade, stabilize the soil, benefit watersheds, and provide a multitude of invaluable environmental benefits (Jose, 2009; Nair et al., 2010).

Crop Modeling

A considerable amount of research has been conducted on assessing the relationship between crop productivity and climate change using simulation models (Challinor et al., 2009; White et al., 2011). GIS-based land-use suitability analysis attempts to identify the most appropriate spatial pattern for future land uses according to specific requirements, preferences, or predictors of some activity (Collins et al., 2001), including suitability of land for agricultural activities (Malczewski, 2004). Numerous crop models currently exist with which to assess crop response to climate change (Challinor et al., 2009; Rivington and Koo, 2011). However, the majority of these models are fine-tuned towards predicting crop yields for global commodity crops (e.g. corn, wheat, soybean, rice). More recently this approach has also been utilized as a method with which to assess crop response to climate change (Lane and Jarvis, 2007; Mathur et al., 2012; Nisar Ahamed et al., 2000; Ramirez-Villegas et al., 2013). GIS-based suitability analysis is quite adaptable to different crop types as long as basic information about the crop's environmental range is known. GIS-based suitability analysis is capable of addressing multiple criteria decision making problems and can incorporate fuzzy logic techniques (Malczewski, 2004). Fuzzy logic (Zadeh, 1965) involves the concept of membership function in which a given

element is numerically represented by the degree to which it belongs to a set. In this way, a measurement within a criterion can have degrees of membership between unsuitable (0) and suitable (1). Because geographical phenomena tend to exhibit continuous spatial variability, Burrough and McDonnell (1998) suggest that fuzzy membership more accurately captures boundaries between land suitability classes then binary or categorical approaches, and such application in crop modelling has been demonstrated more recently (Ramirez-Villegas et al., 2013).

Hawai'i

Hawai'i is one of the many areas where breadfruit, or 'ulu in Hawaiian, was cultivated as a major staple (Meilleur et al., 2014; Lincoln and Ladefoged, 2014; Winter et al., 2018). In traditional times breadfruit was cultivated in a range of cropping systems, from massive arboriculture developments, to mixed agroforestry, to individual and backyard trees (Lincoln and Vitousek, 2017). Following European colonization a dramatic shift away from traditional crops occurred (Kagawa-Viviani et al., 2018), although many pockets of traditional agriculture and associated practices remained (Lincoln et al., 2018b) and remnant trees and production systems persisted (e.g., Lincoln and Ladefoged, 2014; Lincoln *in press*). Over the past 20 years, significant efforts have occurred to revitalize breadfruit in Hawai'i. Such efforts have included large-scale tree giveaways (Lysak, 2018), restoration of traditional agricultural systems (Lincoln et al 2018b), a growing local food producing sector (Langston and Lincoln, 2018), and consumer education such as outreach, chef campaigns and festivals (Ragone et al., 2016). Such initiatives have resulted in significant increases in production at multiple levels including backyard trees, small diversified farm plantings, and larger mono-cropped orchards (Langston and Lincoln, 2018; Lysak, 2018).

In addition to a robust ancient and recent history of breadfruit, Hawai'i has been hailed as a "model system" for ecological investigations (Vitousek 1995). Hawai'i's consistent geology and wide, well-defined variation in climate and substrate age allow for the study of ecology with a degree of precision that cannot be duplicated elsewhere. Environmental gradients in mean annual temperature (from <10-24 C), annual precipitation (from <200->10,000 mm), and substrate age (from hot rock to >4000 kyr) are among the clearest, broadest, and most orthogonal on Earth (Juvik and Juvik 1998). The dense matrix of environmental gradients results in Hawai'i having the densest concentration of ecosystems on the planet (Asner et al., 2005). Thus, Hawai'i is an excellent location to tune habitat distribution models, including for agricultural species and activities.

Purpose

The primary purpose of this paper is to develop an empirically-validated, fuzzy-set model for breadfruit production that incorporates both climate and soil data, and to explore the global potential for breadfruit cultivation in the current and future climate scenarios. This builds upon prior work that conducted a two-tiered suitability model for breadfruit based on rainfall and temperature (Lucas and Ragone, 2012). The developed model is utilized to further assess potential changes in breadfruit production over time with anticipated climate scenarios to understand global, and region specific, changes in productive potential.

2. Methods

Modeling Approach

The crop model methodology in this study utilizes a basic mechanistic approach in which 1) environmental criteria deemed important to the crop's success are selected, 2) suitable environmental ranges for the crop are determined for each of the selected criteria, 3) fuzzy sets

are constructed for each criteria based on the environmental ranges, representing the approximate niche of the crop, and 4) a crop suitability score is calculated based on how closely the current or future environmental conditions match the constructed fuzzy sets, 5) model is validated and refined in an iterative process as required.

The initial environmental ranges for breadfruit were obtained from the EcoCrop database (FAO, 2000) that contains an optimum range and an absolute range for each parameter. Environmental criteria with values within the optimal range represent perfect suitability while values outside the absolute range are considered unsuitable. Fuzzy sets were constructed using the methodology adapted from Ramirez-Villegas et al. (2013) which utilized a linear algorithm to systematically construct the fuzzy set used to derive scores (0-100) between suitable and unsuitable:

$$SUIT_i = \begin{cases} 0, & P_i < ABS_{min} \\ (P_i - ABS_{min})/(OPT_{min} - ABS_{min}) * 100, & ABS_{min} \leq P_i < OPT_{min} \\ 100, & OPT_{min} \leq P_i < OPT_{max} \\ 1 - (P_i - OPT_{max})/(ABS_{max} - OPT_{max}) * 100, & OPT_{max} \leq P_i < ABS_{max} \\ 0, & P_i > ABS_{max} \end{cases}$$

where SUIT is the suitability score, and P is the measured environmental criterion at the site (i.e., each pixel), and ABSmin, ABSmax, OPTmin, and OPTmax are the absolute and optimal environmental ranges for the criterion of a particular crop.

Five environmental criteria were selected to evaluate breadfruit crop suitability. These criteria were rainfall, average temperature, solar radiation, soil drainage, and soil pH. The overall suitability score for each crop was calculated on a per pixel basis using the minimum value of the sets the cell location belongs to:

$$SUIT_{Overall} = \min(C_{SUIT1}, \cdots, C_{SUITn})$$

where *n* is the number of criteria used in the evaluation. This conservative approach is based on the law of the minimum (Paris, 1992), in which crop yield is proportional to the most limiting nutrient; the same idea can be applied to environmental conditions and has been utilized in similar GIS crop-suitability analysis (Bowen and Hollinger, 2002).

Model Development, Refinement and Validation

An initial suitability model was derived for Hawai'i utilizing reported optimal and absolute parameter levels from the EcoCrop database (FAO 2000). Spatial data layers were obtained from the Hawaii Rainfall Atlas (Giambelucca et al., 2013), the Hawaii Evapotranspiration Atlas (Giambelucca et al., 2014) and the SSURGO database (Soil Survey Staff 2018). All calculations were performed in R Studio with a spatial resolution of 50m. Each environmental criterion was evaluated separately producing individual suitability maps, then all maps were combined into a final overall suitability map. This approach allows flexibility within the model and the ability to add new evaluation criteria with minimal changes to the scripting.

Based on previous modeling of breadfruit habitat (e.g., Lucas and Ragone, 2012; Lincoln and Ladefoged 2014), the EcoCrop parameters were known to not be perfectly accurate. We utilized systematic surveys of breadfruit on four islands (Kaua'i, Molokai, O'ahu, and Hawai'i) to map over 1,200 individual trees under rainfed conditions (e.g., Lincoln and Ladefoged 2014; Lincoln *in press*). Using the initial habitat suitability maps for each of the five parameters and the breadfruit tree distributions, a panel of experts were convened to discuss and refine the optimal and absolute levels for each of the parameters. Model was regenerated with the new parameters for two iterations. To validate the new model, yield and productivity data from 56 producer sites were used to conduct linear regressions against the predicted suitability (see Lincoln et al., 2019 for details on sites and methods of producer sites).

Following validation of the model in Hawai'i, the derived ranges of optimal and absolute environmental conditions were used to run the model at a global level. Environmental layers, mean annual temperature, rainfall and solar radiation were acquired from WorldClim (Fick and Hijmans, 2017) and global soil data, ph and drainage class from the WISE database (Batjes 2015, 2016). A comparison of the global model to a previous model developed by Lucas and Ragone (2012) was conducted by spatially overlaying the two datasets and extracting corresponding suitability scores by pixel.

Future projections

Global average annual temperature and rainfall projection data from the WorldClim database were applied to our model to create future suitability scores. These datasets represent the 2060-2080 average using global climate models (GCM) from the CMIP5 of the IPPC Fifth Assessment that were downscaled to 30 arc second (~1km at the equator) spatial resolution using current WordClim 1.4 as baseline current (1950-2000) data (Hijmans 2005). We ran all of the 17 GCMs which had both Representative Concentration Pathway (RCP) 8.5 and 4.5, representing extreme and intermediate Greenhouse Gas scenarios, through our breadfruit model script. Final model outputs were averaged to represent 2070 breadfruit suitability for each RCP. Finally, each RCP scenario was compared to the current global suitability output to determine how much suitability will increase or decrease in the next 50years by spatially overlaying the two datasets and extracting corresponding suitability scores by pixel.

3. Results

Hawai'i Model and Validation

The initial model utilizing parameters derived from EcoCrop proved to be restrictive compared to the distribution of naturalized trees surveyed in Hawai'i (Table 1). In particular, restriction in rainfall and drainage were deemed highly restrictive. For instance, the absolute maximum rainfall provided by EcoCrop was 3,500 mm/yr, but experts provided numerous examples of breadfruit growing substantially wetter areas within the Pacific such as areas of Samoa and Marquesas, and trees were mapped that were growing in naturalized conditions in Hawai'i in substantially wetter areas. Similarly, there were ample trees documented in Hawai'i growing on higher and lower drainage class than reported by EcoCrop. Less significant changes were recommended for temperature and pH, and no changes to solar radiation were suggested. Final parameters were in all cases less restrictive than provided by EcoCrop (Table 1).

Table 1. Final model parameters derived and the initial model parameters obtained from the EcoCrop database.

		Abs Min	Abs Max	Opt Min	Opt Max
**Final Model Parameters**	**Temp** (℃)	17	40	21	33
	**Rain** (mm/yr)	750	8000	1500	4000
	**Solar Rad.** (W/m^2)	20	295	50	197
	**pH**	4	8.7	5	6.5
	**Drainage Class**	2	7	4	6
**EcoCrop Parameters**	**Temp** (℃)	16	40	21	33
	**Rain** (mm/yr)	1000	3500	1500	3000
	**Solar Rad.** (W/m^2)	20	295	50	197
	**pH**	4.3	8.7	5.5	6.5
	**Drainage Class**	4	6	4	6

Following the finalization of parameters, a validation was conducted via linear regression utilizing yield and productivity data from 56 producer sites; data was derived as reported in Lincoln et al., 2019. Of the 56 sites, 11 both irrigated and amended soils, 15 irrigated only, 4 amended soils only, and 27 neither irrigated amended soils. The sites differed in both their

actual productivity and their modeled suitability (Table 2). A linear regression between “natural” sites (no irrigation and no soil amendments) and our overall suitability model demonstrated a strong, significant relationship (r^2 0.86, p<0.001); when all non-irrigated sites (including fertilized sites) are used (n=42), the relationship remains strong (r^2 0.78, p<0.001). As a further check we ran the model excluding rainfall (to simulate irrigated sites), pH (to simulate sites with soil amendment) and both (Table 2). For non-irrigated sites with soil amendments the agreement with the model (excluding the pH layer) was good (r^2 0.73, p<0.0001). For irrigated sites relationships demonstrate marginal significance despite the low number of samples and ranges, with moderately high levels of correlation remaining for both with (r^2 0.54, p<0.0096) and without (r^2 0.86, p<0.0728) soil amendment.

Table 2. Summary of validation site statistics in terms of productivity and modeled suitabilit

Irrigated	Fertilized	n	Productivity	Suitability (All)	Suitability (no pH)	Suitability (no rainfall)	Suitability (no pH&rain)
N	N	27	6.9	63.2	69.6	74.9	85
N	Y	15	7.9	75.7	81.9	86.5	97.9
Y	N	4	8.5	21.2	21.2	59.5	87.5
Y	Y	11	8.7	8.3	8.3	72.1	99.5

Following validation of the model parameters and confidence in its overall accuracy, a final fuzzy set model was produced for Hawai’i (Fig. 1). The effects of temperature and rainfall are clearly visible, with greater potential on the wetter windward (eastern) sides of the islands and near the warmer coasts. The textured breaks in the model represent the soil parameters driven by different aged lava flows on the younger (southern) islands and valley topography on the older (northern) islands.

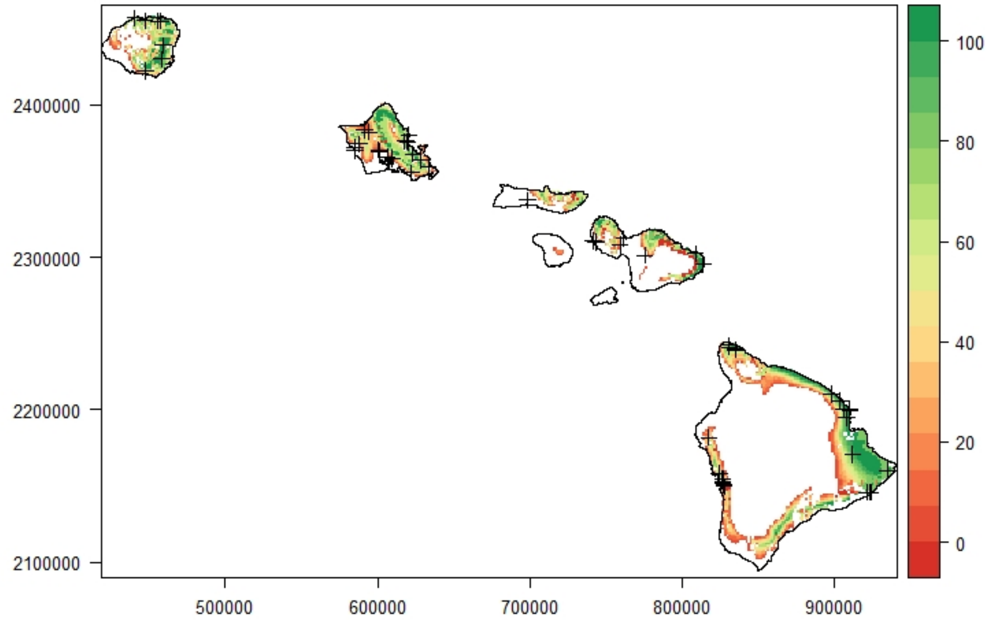

Figure 1. Suitability for productive capacity of breadfruit in Hawai'i, with scores ranging from 0 (white, cannot cultivate) to black (100, ideal cultivation) based on 5 climate and soil parameters. Validation sites are marked by the balck crosshairs.

Global Model and Comparison to Previous Model

Following the development, refinement, and validation of the model using Hawai'i as a model system, the model parameters were applied to global data layers to make global predictions of production potential (Fig. 2).

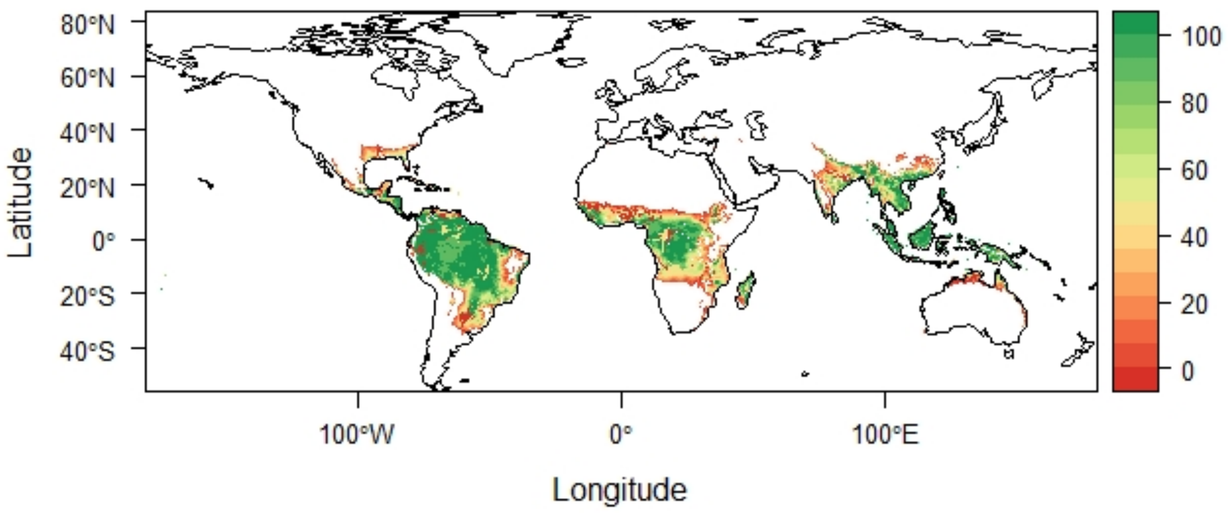

Fig. 2. Global breadfruit suitability extrapolated from the environmental parameters derived in Hawai'i.

We compared our empirically validated model against the only previous global breadfruit suitability model published (Fig. 3), which applied rainfall and temperature to define two classes of breadfruit habitat: "suitable" and "best" (Lucas and Ragone, 2012). Overall our model is more inclusive, with the model by Lucas and Ragone (2012) suggesting ~26,900,000 km^2 of suitable habitat (~14,800,000 km^2 of "best" and ~12,100,000 of "suitable"), and our model suggesting ~35,100,000 km^2 of suitable habitat - an ~30% increase in total cultivable area. This is likely due to our more inclusive thresholds for rainfall and temperature as defined in the model fitting process. Of the lands excluded by Lucas and Ragone (2012) and included by our model, ~70% of those lands score 50 or below on the fuzzy set; however, ~10% do show exceptional suitability, scoring 90 or above. Of the lands included by Lucas and Ragone (2012), our model excludes ~1,300,000 km^2 of "suitable" lands and 700,000 km^2 of "best" lands. A brief examination of excluded pixels indicates that these exclusions primarily resulted from the consideration of soil parameters in our model. Overall, however, the models show fairly good alignment. Of the "best" lands identified by Lucas and Ragone (2012), ~90% of the points

demonstrate suitability values of 90 or above. The mean suitability of all “best” lands is 89, for “suitable” lands 61, and for “unsuitable” lands 0.56.

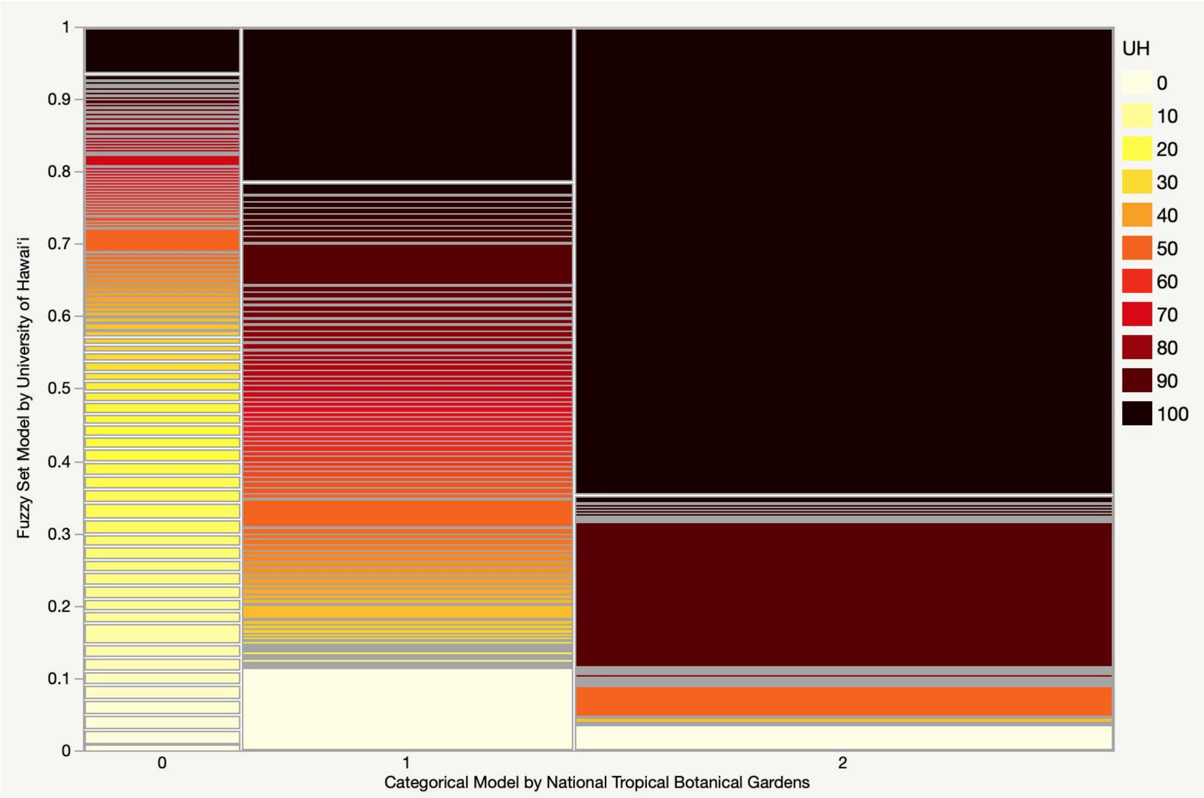

Fig. 3. A mosaic plot comparing a previous global breadfruit model by Lucas and Ragone (2012) on the x-axis in categories of unsuitable (0), suitable (1), and best (2), and our fuzzy set model on the y-axis represented by percentage total of each score.

Future Projections

Breadfruit suitability was assessed using our fuzzy set model using climate projections of precipitation and temperature for the RCP 4.5 and RCP 8.5 climate scenarios (Fig 4). The results demonstrate successive increase in suitability in the RCP 4.5 and RCP 8.5 scenarios compared to the current suitability (Table 3). At all levels of suitability our model predicts an increase of total cultivable land (an increase of 27% and 89% for RCP 4.5 and 8.5 respectively) as well as a total increase in average suitability (an increase of 14% and 45% respectively).

While a large portion of the total increase in habitat occurred under marginal suitability (<20), increases in the cultivable area of all suitability classes is clear.

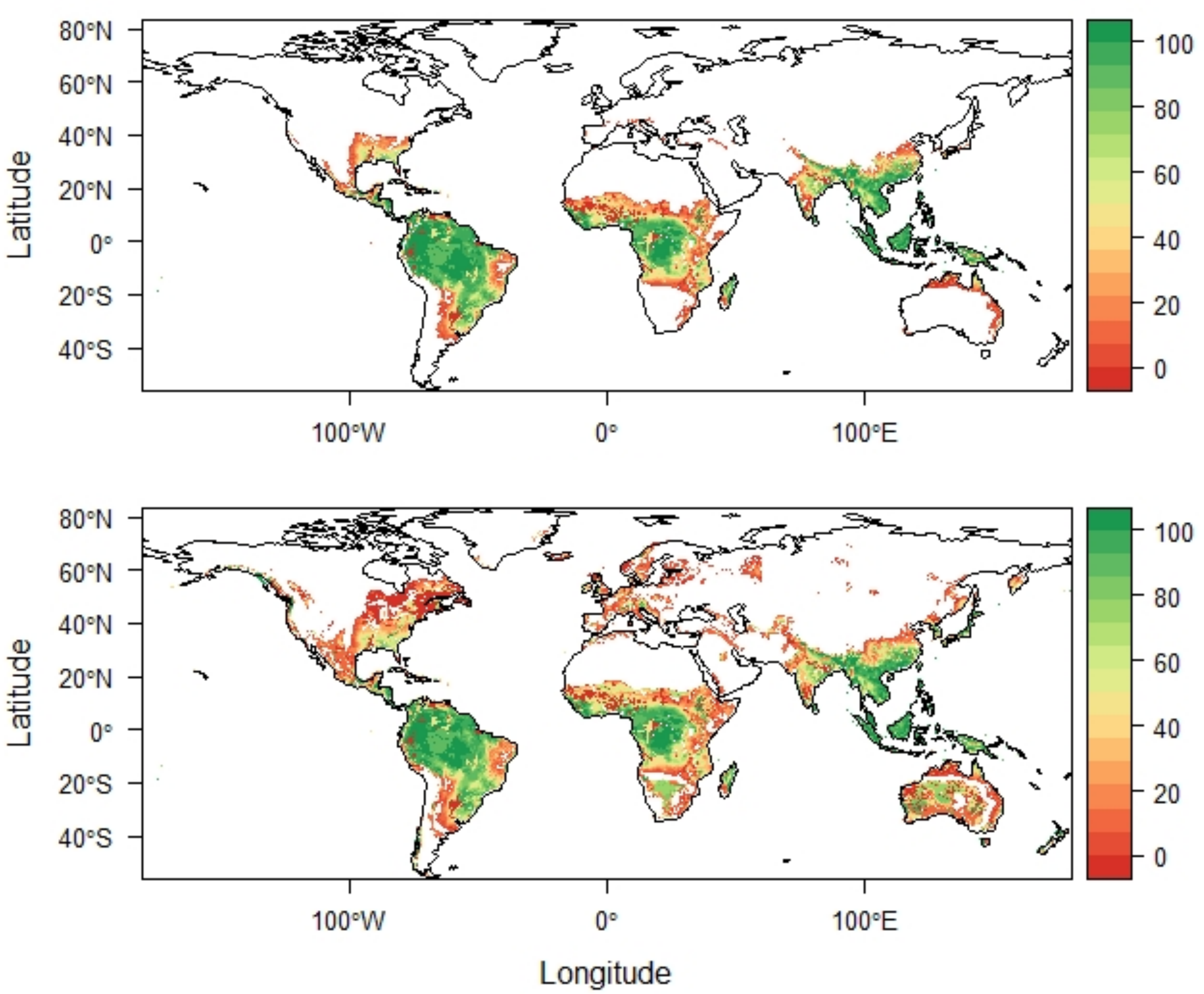

Fig. 4. Modeled future global suitability in 2070 for RCP 4.5 (top) and RCP 8.5 (bottom) utilizing values from 17 GCM models.

Table 3. Area of breadfruit suitability in millions of km2 for current and future climate projections, presented as total and individual bins as discussed.			
	Current	RCP 4.5	RCP 8.5
**Total**	32.66	41.62	61.71
**Bin**			
<1	2.59	3.53	9.35
1 - 10	2.11	4.68	10.38
11 - 20	1.97	3.62	6.20
21 - 30	1.83	2.90	5.28
31 - 40	2.03	2.50	4.62
41 - 50	3.27	3.74	5.17
51 - 60	1.64	1.90	3.16
61 - 70	1.42	1.96	2.93
71 - 80	1.63	2.12	4.17
81 - 90	4.25	5.25	6.22
91 - 100	12.50	12.94	13.59
**Group**			
<1 TO 30	8.51	14.73	31.21
31 TO 70	8.36	10.11	15.88
71 TO 100	18.38	20.31	23.97

While our model suggests a net increase in total suitable land and general suitability over the next 50 years, the changes are not uniform and some areas do show a decrease in suitability, including total loss of cultivation (Fig. 5). In particular, losses in suitability occur in currently suitable areas of Central and South America, while large gains are seen in southeast Asia, southeastern United States, and southeastern parts of South America. In general though, currently productive areas remain largely unchanged. Under the 4.5 scenario, the area that does not change is 43% of the total modelled area (~16,700,000 km^2), and the area that increases/decreases by a suitability of up to 10 is a further 35%, making the total area that remains unchanged or minimally unchanged 78% of the total modeled area (~27,400,000 km^2). Under the RCP 8.5 climate scenario, the area that will remain unchanged over the next 50 years is 43% of the totaled modelled area (~15,200,000 km^2). The area that will minimally change (+/-

10 suitability score), will be another 30%, to make the total area unchanged or minimally unchanged 73% of the total modeled area (~25,500,000 km^2).

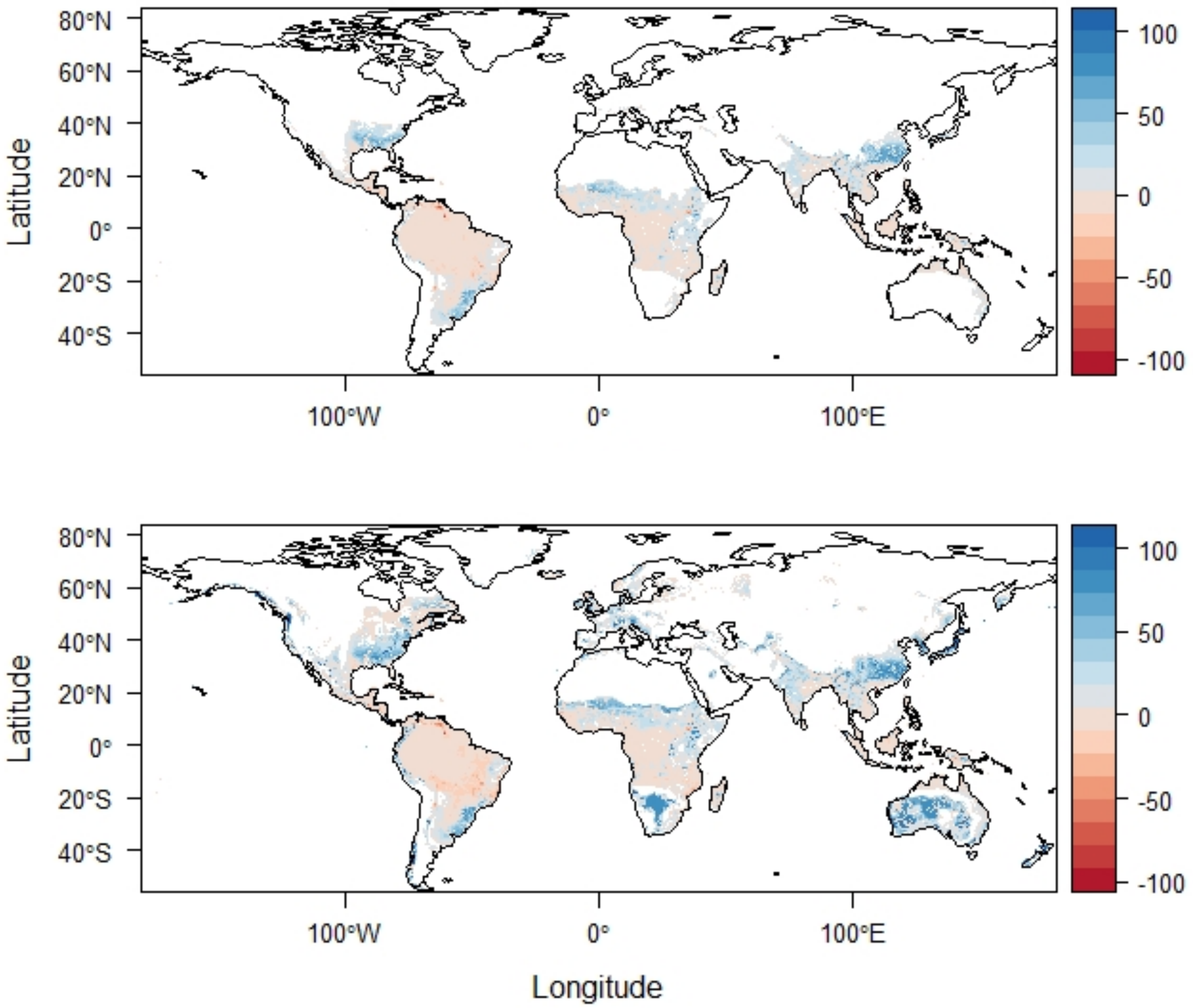

Fig 5. Changes (increase/decrease) in breadfruit suitability over the next 50 years under (a) RCP 4.5 and (b) RCP 8.5.

4. Discussion

The current opportunity for breadfruit is substantial, with, in general, large increases in total cultivable area and average suitability of that area under climate change projections. This is encouraging given that many of our current staple crops, particularly those grown extensively in temperate regions, are expected to decline in suitability with projected future climate scenarios. Furthermore, most currently producing regions are not negatively affected, providing some security and stability in the face of projected changes.

The vast growth in very low suitability in our model is a facet of outlier GCM models. All of the very low (<1) suitability numbers result from pixels where only one of the 17 GCMs indicated conditions suitable for breadfruit. Further examination into the variability would provide stronger confidence regarding future habitat and thereby mitigate risk in investment. This is particularlly important for a neglected crop species as there is already substantial risk because of the large scale-lack in agronomic research, post-harvest research, and market development.

As mentioned, our model indicates expansion of area in all bins of suitability. In our observation of validation sites we observed that suitability of 30 or below represented very poor producing trees - the plants would grow and bear fruit but only on the order of 10% of what trees in very high suitability were able to produce. In looking at these projected suitability then we feel it would be wise to exclude the sites with suitability scores <30. Similarly at the upper end of the spectrum, site with suitability of >70 appear to all be highly productive, with only slight changes in relatively high yields. Therefore, a more tempered approach might be to think of “moderate” (31-70) and “high” (71-100) quality habitat for breadfruit. Such an approach would also inherently eliminate the vast areas of very low suitability (<10) caused by model outliers. Applying this breakdown (Table 3), we see a 10% and 30% increase in high suitability sites for RCP 4.5 and RCP 8.5 respectively, and a corresponding increase of 21% and 90% in moderate suitability.

A model shortcoming is that average annual data was used. This may be particularly problemsome in areas that get below freezing temperatures, as frost has been reported to kill breadfruit trees. While the absolute minimum average annual temperature was set at 17 ℃, freezing temperatures for short periods of time could still occur and be offset by much higher temperatures. Any future modeling efforts should certainly take an approach that considers monthly extremes as another model parameter. However, for the tropical and subtropical regions of the world this shortcoming is not expected to impact the results. Other extreme events, such as heat waves, floods, hurricanes, etc. are also not considered in examining suitability in this way.

Given such dramatic increases in global suitability, our results made us wonder what other tropical crops may flourish under future climate conditions. In planning for future adaptation it would make sense to identify and nurture these crops now to increase their planting, as well as the technical and infrastructural support needed. More subtly, in working with breadfruit extensively there is also a strong need to shift consumer preferences, grow peoples' pallette for new crops, change perception and markets, and so on. Bringing an underutilized crop into the market as a major food source is a rare and difficult task that face many hurdles, and only a handful of case-study examples exist (such as, I would argue, quinoa).

5. Conclusion

Much more attention needs to be given to neglected and underutilized crop species as a critical component for addressing mounting issues of food security and nutrition in a changing world.

6. Acknowledgements

This work is supported by the USDA National Institute of Food and Agriculture HATCH [project 8035-H] and McIntire-Stennis (project 8038M-MS) managed by the College of Tropical Agriculture and Human Resources, and the Western SARE Research and Education program [grant number SW17-050].

7. Statement

The authors declare that this work is their own, and represents no conflicts of interest or financial ties to the research. No funding agencies influenced or directed this work. Data script and model outputs will be made available upon request.

8. References

Asner, G. P., Elmore, A. J., Hughes, R. F., Warner, A. S., and Vitousek, P. M. (2005) ‘Ecosystem structure along bioclimatic gradients in Hawaii from imaging spectroscopy’, *Remote Sensing of Environment* 96(3-4), pp497-508

Bacon, C.M., Sundstrom, W.A., Gómez, M.E. F., Méndez, V.E., Santos, R., Goldoftas, B., Dougherty, I. 2014. Explaining the ‘Hungry Farmer Paradox’: Smallholders and Fair Trade Cooperatives Navigate Seasonality and Change in Nicaragua’s Corn and Coffee Markets. *Global Environmental Change* 25 (1): 133–49. doi:10.1016/j.gloenvcha.2014.02.005.

Batjes, N. H. (2015). *World soil property estimates for broad-scale modelling (WISE30sec)* (No. 2015/01). ISRIC-World Soil Information.

Batjes, N. H. (2016). Harmonized soil property values for broad-scale modelling (WISE30sec) with estimates of global soil carbon stocks. *Geoderma*, *269*, 61-68.

Bell, J., and M. Taylor. 2015. “Building climate-resilient food systems for Pacific Islands.” WorldFish. Program Rep: 2015-15. Penang, Malaysia.

Bowen, R.C. and Hollinger, S.E., 2002. Alternative Crops Web Site, Illinois State Water Survey. Champaign Illinois, http://www.sws.uiuc.edu/data/altcrops/.

Burrough, P.A. and McDonnell, R., 1998. Principles of geographical information systems, 333. Oxford university press Oxford.

Buyck, M. 1993. The international community's commitment to combating micronutrient deficiencies. *Journal of Food Nutrition and Agriculture* 7: 2-7.

Challinor, A., Wheeler, T., Craufurd, P. and Slingo, J., 2005. Simulation of the impact of high temperature stress on annual crop yields. Agricultural and Forest Meteorology, 135(1): 180-189.

Collins, W.W. and Hawtin, G.C. 1999. Conserving and Using Crop Plant Biodiversity in Agroecosystems. Pp. 267-281 in Biodiversity in Agroecosystems (W.W. Collins and C.O. Qualset, eds). CRC Press, Boca Raton, Washington.

FAO. 1996. Global plan of action for the conservation and sustainable utilisation of plant genetic resources for food and agriculture. Leipzig, Germany, June 1996.

FAO. 1999. Agricultural Biodiversity, Multifunctional Character of Agriculture and Land Conference, Background Paper 1. Maastricht, Netherlands. September 1999.

FAO. 2000. Crop Ecological Requirements Database (ECOCROP). http://ecocrop.fao.org/ecocrop/srv/en/home

FAO. 2009. “International Treaty on Plant Genetic Resources for Food and Agriculture,” 56. http://www.fao.org/3/a-i0510e.pdf.

FAO, IFAD, UNICEF, WFP and WHO. 2017. The State of Food Security and Nutrition in the World 2017. Building resilience for peace and food security. Rome, FAO

FAO. 2018. Biodiversity: Plants. Retrieved June 26, 2018, from http://www.fao.org/biodiversity/components/plants/en/

Fick, S.E. and R.J. Hijmans, 2017. Worldclim 2: New 1-km spatial resolution climate surfaces for global land areas. *International Journal of Climatology*.

Giambelluca, T.W., Q. Chen, A.G. Frazier, J.P. Price, Y.L. Chen, P.S. Chu, J.K. Eischeid, and D.M. Delparte. 2013. Online rainfall atlas of Hawai‘i. Bull. of the Am. Meteorol. Soc. 94(3):313-316. http://dx.doi.org/10.1175/BAMS-D-11-00228.1

Giambelluca, T.W., X. Shuai, M.L. Barnes, R.J. Alliss, R.J. Longman, T. Miura, Q. Chen, A.G. Frazier, R.G. Mudd, L. Cuo, and A.D. Businger. 2014. Evapotranspiration of Hawaii. Final report submitted to the U.S. Army Corps of Engineers-Honolulu District, and the Commission on Water Resource Management, State of Hawai‘i, USA.

Godfray, H. C. J., Beddington, J. R., Crute, I. R., Haddad, L., Lawrence, D., Muir, J. F., ... & Toulmin, C. (2010). Food security: the challenge of feeding 9 billion people. *science*, *327*(5967), 812-818.

Hijmans, R.J., S.E. Cameron, J.L. Parra, P.G. Jones and A. Jarvis, 2005. Very high resolution interpolated climate surfaces for global land areas. International Journal of Climatology 25: 1965-1978

Jones, A. M. P., Ragone, D., Aiona, K., Lane, W.A., and Murch, S.J. 2011. “Nutritional and Morphological Diversity of Breadfruit (Artocarpus, Moraceae): Identification of Elite Cultivars for Food Security.” *Journal of Food Composition and Analysis* 24 (8). Elsevier Inc.: 1091–1102. doi:10.1016/j.jfca.2011.04.002.

Jones, A. M.P., Murch, S.J., Wiseman, J. and Ragone, D. 2013. “Morphological Diversity in Breadfruit (Artocarpus, Moraceae): Insights into Domestication, Conservation, and Cultivar Identification.” *Genetic Resources and Crop Evolution* 60 (1). doi:10.1007/s10722-012-9824-8.

Jose, S. (2009). Agroforestry for ecosystem services and environmental benefits: an overview. *Agroforestry systems*, *76*(1), 1-10.

Juvik, S. P. and Juvik, J. O. (1998) *Atlas of Hawaii*, University of Hawaii Press, Honolulu, HI

Kagawa-Viviani, A., Levin, P., Johnson, E., Kantar, M., Ooka, J., Baker, J.D., and Lincoln, N.K. (2018). I ke ēwe ‘āina o ke kupuna: Hawaiian ancestral crops in perspective. *Sustainability* 10(12), 4607.

Lane, A. and Jarvis, A., 2007. Changes in climate will modify the geography of crop suitability: agricultural biodiversity can help with adaptation. Journal of Semi-arid Tropical Agricultural Research, 4(1): 1-12.

Langston, B., and Lincoln, N. (2018). The role of breadfruit in biocultural restoration and sustainability in Hawai‘i. *Sustainability*, 10(11), 3965.

Lincoln, N.K. (in press). Pākukui: the productive fallow system of Hāmākua, Hawai’i. In Cairns, M [ed], *Farmer innovation and best practices by shifting cultivators in Asia-Pacific.* CABI Press: Oxfordshire, UK.

Lincoln, N.K., and T.N. Ladefoged. 2014. Agroecology of pre-contact Hawai'ian dryland farming: the spatial extent, yield and social impact of Hawai'ian breadfruit groves in Kona, Hawai'i. J. Archaeol. Sci. 49:192–202.

Lincoln, N. K., and Vitousek, P. M. (2017). Indigenous Polynesian Agriculture in Hawaiʻi. *Oxford Research Encyclopedias: Environmental Science* March (2017). DOI: 10.1093/acrefore/9780199389414.013.376

Lincoln, N.K., D. Ragone, N. Zerega, L.B. Roberts-Nkrumah, M. Merlin, and A.M Jones. (2018a). Grow us our daily bread: A review of breadfruit cultivation in traditional and contemporary systems. *Horticultural Reviews*. Vol. 46: 299-384.

Lincoln, N.K., Rossen, J., Vitousek, P., Kahoonei, J., Shapiro, D., Kalawe, K., Pai, M., Marshall, K., and Meheula, K. (2018b). Restoration of ʻāina maloʻo on Hawaiʻi Island: Expanding Biocultural Relationships. *Sustainbility*, 10(11), 3985.

Lincoln, N.K., Radovich, T., Acosta, K., Isele, E., Cho, A. (2019). Towards standardization of foliar tissue sampling of breadfruit. *Hort. Tech.* 29(4), 443-449

Lysak, M. Improving Food and Nutrition Security through Exploration of Market Potentials by Revitilizgin Breadfruit as a Strategy for Adapting to Climate Change. Ph.D. Thesis, Department of Plant and Environmental Science, University of Copenhagen, Copenhagen, Denmark, 2018.

Mabhaudhi, T., Chibarabada, T. Chimonyo, V. G. P.and Albert Modi. 2016. Water-Food-Nutrition-Health Nexus: Linking Water to Improving Food, Nutrition and Health in Sub-Saharan Africa. *International Journal of Environmental Research and Public Health* 13 (1). Multidisciplinary Digital Publishing Institute: 107. doi:10.3390/ijerph13010107.

Malczewski, J., 2004. GIS-based land-use suitability analysis: a critical overview. Progress in Planning, 62(1): 3-65.

Mathur, P., Ramirez-Villegas, J. and Jarvis, A., 2012. The Impacts of Climate Change on Tropical and Sub-tropical Horticultural Production. Tropical Fruit Tree Species and Climate Change: 27.

McGregor, A.M., L. Tora and V. Lebot. 2014. “Planting breadfruit orchards as a climate change mitigation strategy for the Pacific islands.” Paper presented at XXIX Intl Hort Congress. Brisbane.

Meilleur, B.A., R.R. Jones, C.A. Titchenal, and A.S. Huang. 2004. Hawaiian Breadfruit: Ethnobotany, Nutrition, and Human Ecology. CTAHR, University of Hawaii, Honolulu, HI.

Nair, P. R., Nair, V. D., Kumar, B. M., & Showalter, J. M. (2010). Carbon sequestration in agroforestry systems. In *Advances in agronomy* (Vol. 108, pp. 237-307). Academic Press.

Nisar Ahamed, T., Gopal Rao, K. and Murthy, J., 2000. GIS-based fuzzy membership model for crop-land suitability analysis. Agricultural Systems, 63(2): 75-95.

Padulosi, S., Hodgkin, T., Williams, J. T., and Haq, N. 2002. “Underutilized Crops: Trends, Challenges and Opportunities in the 21st Century.” *Managing Plant Genetic Diversity. Proceedings of an International Conference, Kuala Lumpur, Malaysia, 12-16 June 2000*, no. December 2014: 323–38. doi:10.1079/9780851995229.0323.

Padulosi, S., Hodgkin, T., Williams, J. T., & Haq, N. (2002). Underutilized crops: trends, challenges and opportunities in the 21st century, managing plant genetic diversity. *CAB International, Wallingford*.

Padulosi, S., Thompson, J. and Rudebjer, P. 2013. *Fighting Poverty, Hunger and Malnutrition with Neglected and Underutilized Species: Needs, Challenges and the Way Forward*. *Bioversity International*. www.bioversityinternational.org.

Paris, Q., 1992. The Return of von Liebig's "Law of the Minimum". Agronomy Journal, 84(6): 1040-1046.

Porter, J.R., Xie, L., Challinor, A.J., Cochrane, K., Howden, S.M., Iqbal, M.M., Lobell, D.B., Travasso, M.I. 2014. "Food Security and Food Production Systems." *Climate Change 2014: Impacts, Adaptation, and Vulnerability. Part A: Global and Sectoral Aspects. Contribution of Working Group II to the Fifth Assessment Report of the Intergovernmental Panel on Climate Change*. doi:10.1111/j.1728-4457.2009.00312.x.

Porter, J. R., Howden, M., & Smith, P. (2017). Considering agriculture in IPCC assessments. *Nature Climate Change*, *7*(10), 680.

Prescott-Allen, R.., & Prescott-Allen, C.. (1990). How many plants feed the world?. *Conservation Biology*, *4*(4), 365-374.

Ragone, D. 1997. "Breadfruit (Artocarpus Altilis (Parkinson) Fosberg Promoting the Consevation Ans Use of Underutilized and Neglected Crops 10." *International Pant Genetic Resources Institute*, 77. http://medcontent.metapress.com/index/A65RM03P4874243N.pdf.

Ragone, D. and Lucas, M.P. 2012. "Will Breadfruit Solve the World Hunger Crisis? New Developments in an Innovative Food Crop." *ArcNews*. http://www.esri.com/news/arcnews/summer12articles/will-breadfruit-solve-the-world- hunger-crisis.html.

Ragone, D.; Elevitch, C.; Dean, A. Revitalizing breadfruit in Hawaii—A model for encouraging the cultivation and use of breadfruit in the tropics. *Trop. Agric.* **2016**, *93*, 213–224.

Ramalingaswami, V. 1995 New global perspectives on overcoming malnutrition. *American Journal of Clinical Nutrition* 61: 259-263.

Ramirez-Villegas, J., Jarvis, A. and Laderach, P., 2013. Empirical approaches for assessing impacts of climate change on agriculture: The EcoCrop model and a case study with grain sorghum. Agricultural and Forest Meteorology, 170: 67-78.

Rivington, M. and Koo, J., 2011. Report on the meta-analysis of crop modelling for climate change and food security survey, CGIAR Research Program on Climate Change, Agriculture, and Food Security.

Soil Survey Staff, Natural Resources Conservation Service, United States Department of Agriculture. (2018) Soil Survey Geographic (SSURGO) Database. Available online at https://sdmdataaccess.sc.egov.usda.gov. Accessed [month/day/year].

Sraffa, P. 2005. The laws of returns under competitive conditions, p. 216–233. In: G.Z. Sun (ed.). Readings in the economics of the division of labor: The classical tra- dition. Vol. 2. World Sci. Publ., Singapore.

Turi, C. E., Liu, Y., Ragone, D., and Murch, S.J. 2015. Breadfruit (Artocarpus Altilis and Hybrids): A Traditional Crop with the Potential to Prevent Hunger and Mitigate Diabetes in Oceania. *Trends in Food Science & Technology* 45 (2). Elsevier Ltd: 264–72. doi:10.1016/j.tifs.2015.07.014.

United Nations. 2015. “Sustainable Development Goals.” https://www.un.org/sustainabledevelopment/sustainable-development-goals/.

Vitousek, Peter M. 1995 The Hawaiian Islands as a Model System for Ecosystem Studies. Pacific Science 49:2–16.

White, J.W., Hoogenboom, G., Kimball, B.A. and Wall, G.W., 2011. Methodologies for simulating impacts of climate change on crop production. Field Crops Research, 124(3): 357-368.

Willcox, O.W. 1954. Quantitative agro- biology: III. The Mitscherlich equation and its constants. Agron. J. 46:323–326.

Winter, K.B., Lincoln, N.K., and Berkes, F. (2018). The socio-ecological keystone concept: A quantifyable metaphor for understanding the structure, function, and resilience of a biocultural system. *Sustainability*, 10(9), 3294.

Zadeh, L.A., 1965. Fuzzy sets. Information and control, 8(3): 338-353.

Zerega, N. J. C, Ragone, D., and Motley, T.J. 2004. Complex Origins of Breadfruit (Artocarpus Altilis , Moraceae): Implications for Human Migrations in Oceania. *American Journal of Botany* 91 (5): 760–66.
